# Prognostic Factors of In-Hospital Mortality in Patients with Acute Myocardial Infarction Complicated by Cardiogenic Shock

**DOI:** 10.3390/life12101672

**Published:** 2022-10-21

**Authors:** Takanori Sato, Yuichi Saito, Sakuramaru Suzuki, Tadahiro Matsumoto, Daichi Yamashita, Kan Saito, Shinichi Wakabayashi, Hideki Kitahara, Koichi Sano, Yoshio Kobayashi

**Affiliations:** 1 Department of Cardiovascular Medicine, Chiba University Graduate School of Medicine, Chiba 260-8670, Japan; 2 Department of Cardiovascular Medicine, Eastern Chiba Medical Center, Togane, Chiba 283-8686, Japan

**Keywords:** acute myocardial infarction, cardiogenic shock, percutaneous coronary intervention

## Abstract

Among patients with acute myocardial infarction (MI) complicated by cardiogenic shock (CS), in-hospital mortality remains high. In the present study, we aimed to identify factors associated with clinical outcomes of acute MI patients with CS in a contemporary setting. A total of 1102 patients with acute MI undergoing primary percutaneous coronary intervention were included, among whom 196 (17.8%) were complicated by CS. The primary outcome was all-cause death during hospitalization, and factors associated with in-hospital mortality were explored in patients with acute MI and CS. Of the 196 patients with acute MI complicated by CS, 77 (39.3%) died during hospitalization. The rates of non-ST-segment elevation MI (NSTEMI) (33.8% vs. 19.3%, *p* = 0.02) and culprit lesion in the left main or left anterior descending coronary artery (68.8% vs. 47.9%, *p* = 0.004) were higher, while left ventricular ejection fraction (LVEF) was lower (24.4 ± 11.7% vs. 39.7 ± 13.8%, *p* < 0.001) in non-survivors than in survivors. Multivariable analysis identified NSTEMI presentation and lower LVEF as independent predictors of in-hospital death. In conclusion, NSTEMI and low LVEF were identified as factors associated with higher in-hospital mortality. The identification of even higher-risk subsets and targeted therapeutic strategies may be warranted to improve survival of patients with acute MI and CS.

## 1. Introduction

Owing to recent advances in pharmacological therapy and early reperfusion strategies, especially with primary percutaneous coronary intervention (PCI), short-term mortality of acute myocardial infarction (MI) during hospitalization or at 30 days has considerably decreased in the past decades, from roughly 30% in the 1960s to around 5% in the 2010s [1,2,3]. However, even in the current era, mortality of patients with acute MI complicated by cardiogenic shock (CS) remains high. CS immediately after acute MI occurs in approximately 10% and is associated with 30-day mortality of 40–50% [4]. Emergency revascularization of the culprit coronary lesion is the mainstay of treatment in patients with acute MI and CS, and it is the only therapy that has been shown to reduce mortality in a randomized trial setting [5]; no other potential therapeutic strategies, including pharmacological treatment, immediate multivessel PCI, and mechanical circulatory support (MCS), have been established to improve clinical outcomes of this catastrophic condition [4]. Although the clinical evidence that MCS devices can improve outcomes in patients with acute MI and/or CS is lacking, intra-aortic balloon pump (IABP), extracorporeal membrane oxygenation, and intravascular microaxial left ventricular assist device (Impella, Abiomed Inc, Danvers, USA) have been persistently and increasingly used in daily clinical practice [6]. For example, the randomized IABP-SHOCK II trial showed that IABP did not reduce all-cause mortality at 30 days and at long-term follow-up [7,8]. In addition, recent observational studies indicated that the use of Impella rather than IABP was associated with increased medical cost and risks of mortality, major bleeding events, and kidney replacement therapy in patients with acute MI and CS [9,10]. In this context, the identification of even higher-risk subsets may aid in decision making on therapeutic strategies to improve prognosis among revascularized acute MI patients [11]. In previous studies, several clinical factors have been suggested to stratify patient risks in a setting of acute MI complicated by CS, including older age, female gender, impaired cardiac and kidney function, and others [12]. However, recent data on this life-threatening condition are scarce. The purpose of this study was to explore factors associated with in-hospital outcomes of acute MI patients with CS undergoing PCI in a contemporary setting.

## 2. Materials and Methods

### 2.1. Study Population

We conducted a retrospective, bicenter registry study at 2 tertiary referral hospitals, Chiba University Hospital and Eastern Chiba Medical Center [13,14,15,16,17,18]. From January 2012 to March 2020, a total of 1102 patients with acute MI including ST-segment elevation MI (STEMI) and non-ST-segment elevation MI (NSTEMI) underwent primary PCI. No exclusion criteria were applied, and all patients were included in the present analysis. Acute MI was defined according to the fourth universal definition of MI [19]. Primary PCI was performed in all patients per local standard practice, with predominant use of radial access, intracoronary imaging, and new-generation drug-eluting stents [20,21,22,23,24]. All patients provided written informed consent for the examination and PCI procedures, and informed consent for the present study was obtained in the form of opt-out. The ethical committees of Chiba University Hospital and Eastern Chiba Medical Center approved this study.

### 2.2. Definitions

In the present study, patients were divided into 2 groups according to the presence or absence of CS on admission. CS was defined as systolic blood pressure <90 mm Hg and signs of tissue hypoperfusion, described as Killip class 4 [25]. Patients were considered as having CS when inotropes/vasopressors and MCS were needed to maintain systolic blood pressure ≥90 mm Hg. Left ventricular ejection fraction (LVEF) and laboratory data including hemoglobin and creatinine levels were evaluated on admission. Conventional risk factors including hypertension, diabetes, dyslipidemia, and current smoking status were defined by the Japanese Association of Cardiovascular Intervention and Therapeutics’ criteria [26]. Hypertension was defined as a previous diagnosis of hypertension or previous antihypertensive medications, or a new diagnosis of hypertension during hospitalization with systolic blood pressure ≥140 mm Hg and/or diastolic blood pressure ≥90 mm Hg. Diabetes was defined as a previous diagnosis of diabetes or previous glucose-lowering medications, or hemoglobin A1c ≥6.5% on admission. Dyslipidemia was defined as low-density lipoprotein cholesterol ≥140 mg/dL, high-density lipoprotein cholesterol <40 mg/dL, or fasting triglycerides >150 mg/dL, or a previous diagnosis of dyslipidemia. Low- and high-density lipoprotein cholesterol levels were evaluated in a fasting or non-fasting state. Current smoking in this study was defined as a history of smoking within the past year [26].

### 2.3. Outcomes and Statistical Analysis

The primary outcome was all-cause death during the index hospitalization for acute MI, which was obtained from medical records at Chiba University Hospital and Eastern Chiba Medical Center. The primary outcome was captured in all patients. The primary interest of the present analysis was factors associated with in-hospital mortality in patients with acute MI and CS undergoing primary PCI. Statistical analysis was performed with JMP Pro 15.0.0 software (SAS Institute, Cary, USA). All data are expressed as mean ± standard deviation or median (interquartile range) for continuous variables and as number (percentage) for categorical variables. Continuous variables were compared with Student’s *t*-test, and categorical variables were assessed with Fisher’s exact test. Odds ratios and 95% confidence intervals for the risk of in-hospital death were estimated with univariable and multivariable analyses using a logistic regression model. Associated factors with *p* < 0.05 on univariable analyses were included in a multivariable model with age, sex, and body mass index (irrespective of *p* values on univariable analyses). The receiver operating characteristics (ROC) curve analysis was performed based on in-hospital death, and the best cut-off value was established by finding the value that corresponded to the maximum average sensitivity and specificity. A value of *p* < 0.05 was considered statistically significant.

## 3. Results

Of the 1102 patients with acute MI undergoing primary PCI, 196 (17.8%) presented with CS. Patients complicated by CS were more likely to present with STEMI and had a higher rate of left main or left anterior descending coronary artery as the culprit vessel as compared to those without CS (Table S1). During the median length of hospital stay of 22 days *[10,39]*, 77 out of 196 (39.3%) patients died. Table 1 lists baseline patient characteristics of patients with acute MI complicated by CS. Approximately three-quarters of patients presented with STEMI, and cardiac arrest was observed in 59.7% (Table 1). In this study population, 3 (1.5%) patients had cardiac rupture and 3 (1.5%) had ventricular septal perforation.

Table 2 shows procedural characteristics. Intravascular ultrasound, drug-eluting stents, and MCS were used in 98.0%, 87.2%, and 56.6%, respectively (Table 2). Post-PCI Thrombolysis in Myocardial Infarction flow grade of 2 or 3 was achieved in 98.4%. Among patients complicated by CS, patients developing in-hospital death were more likely to present with NSTEMI and be treated with MCS and had impaired renal function and lower LVEF as compared with survivors (Table 1 and Table 2). Multivariable analysis identified NSTEMI presentation and lower LVEF as independent predictors of in-hospital death (Table 3).

**Figure 1:**
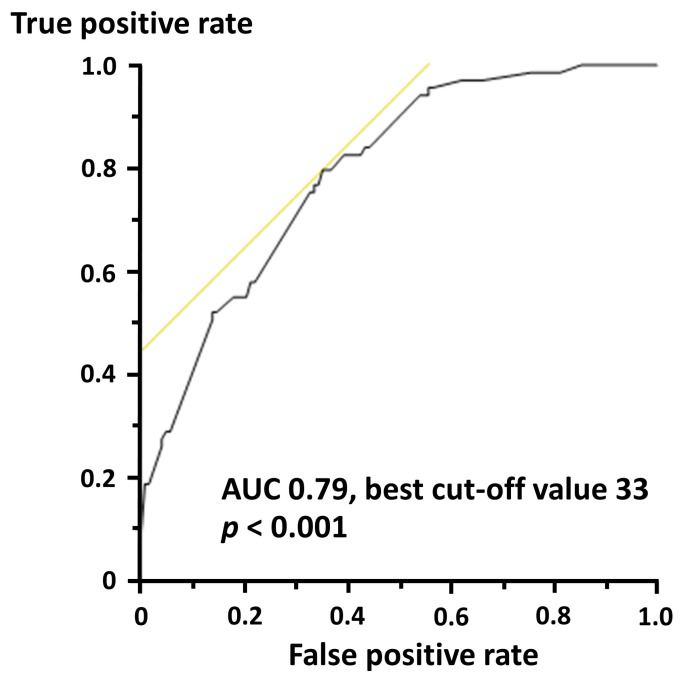
ROC curve for in-hospital mortality. AUC, area under the curve.

**Figure 2:**
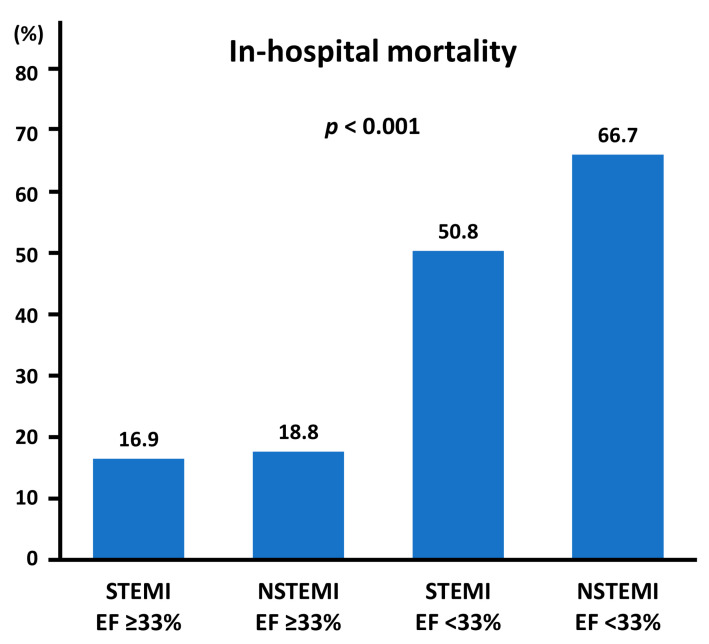
In-hospital mortality stratified by types of MI and EF categories. EF, ejection fraction; MI, myocardial infarction; NSTEMI, non-ST-segment elevation myocardial infarction; STEMI, ST-segment elevation myocardial infarction.

In the ROC curve analysis, LVEF was significantly predictive for in-hospital mortality, with the area under the curve of 0.79 and the best cut-off value of 33% (*p* < 0.001) (Figure 1). With types of MI and LVEF categories with the cut-off value, in-hospital mortality rates were stratified in a stepwise manner (Figure 2).

## 4. Discussion

The present study reinforced that in a contemporary cohort of acute MI patients undergoing primary PCI, nearly 40% of patients died during hospitalization when complicated by CS. Multivariable analysis indicated that NSTEMI presentation and lower LVEF were associated with an increased risk of in-hospital death. When patients with acute MI and CS had NSTEMI presentation and LVEF <33% on admission, in-hospital mortality reached up to two-thirds.

### 4.1. Type of MI and Cardiogenic Shock

CS is one of the most serious complications of acute MI, which is characterized by low systemic perfusion caused by cardiac pump failure, often leading to multiple organ impairment and poor prognosis [12]. Acute MI is the most common scenario of CS, and acute coronary syndrome (ACS) as an etiology of CS is a predictor of poor prognosis [27]. A multicenter prospective registry in European countries showed that among patients with CS, ACS accounted for more than 80% of etiologies [27]. In the European registry, multivariable analysis identified ACS etiology as a factor significantly associated with in-hospital mortality (adjusted odds ratio 7.4, 95% confidence interval 1.9–29.8) [27]. Even in the current era, mortality of acute MI patients with CS remains considerably high [4]. Although there are significant differences in patient characteristics among types of clinical studies investigating acute MI with CS, 30-day mortality was reported to be nearly 40% in randomized trials and over 45% in real-world registries [28]. Emergency coronary revascularization is a therapeutic strategy established by the randomized SHOCK trial, in which patients with CS due to left ventricular failure complicating acute MI were randomly assigned to emergency revascularization or initial medical treatment [5]. The SHOCK trial had a relatively small sample size (*n* = 302) and was initially reported as a negative study [29]. In the original SHOCK results, overall mortality did not differ significantly between the revascularization and medical therapy groups (46.7% vs. 56.0%, *p* = 0.11) [29], while the long-term follow-up data showed the superiority of emergency revascularization over initial medical treatment with respect to mortality at one year (53.3% vs. 66.4%, *p* < 0.03) [5]. However, no other treatment options have been shown to be beneficial with robust evidence. A comprehensive understanding of risk factors in this specific population may better identify a patient trajectory and aid in managing therapeutic strategies such as pharmacological treatment, mode of revascularization, and MCS use, presumably resulting in improved outcomes and appropriate resource utilization in patients with acute MI complicated by CS [11]. In this context, the identification of prognostic factors ideally available on admission may be clinically relevant.

Although several scoring systems have been proposed to stratify patient risks in a setting of acute MI with CS [12], the impact of types of MI (i.e., STEMI and NSTEMI) remains uncertain. In the United States, an analysis from the National Cardiovascular Data Registry indicated that NSTEMI patients with CS had higher in-hospital mortality than STEMI patients complicated by CS (40.8% vs. 33.1%) [30]. In this nationwide registry, NSTEMI patients with CS were likely to be older and have more comorbidities, including renal impairment and anemia, but less likely to receive PCI than STEMI patients with CS [30]. The present study demonstrated that even in a cohort of patients undergoing PCI, NSTEMI was identified as a factor associated with higher in-hospital mortality. Interestingly, patients complicated by CS were more likely to present with STEMI (Table S1), while STEMI presentation did not lead to poor prognosis among patients with CS. It is well known that NSTEMI patients accompanied by CS are likely to have extensive coronary artery disease including three vessel and/or left main disease, potentially affecting the present study results [30,31], although these coronary characteristics were included in the multivariable model. Because the presence or absence of ST-segment elevation is readily available even in an emergency setting, risk stratification with NSTEMI presentation may be useful in daily practice. Given that the relative prevalence of NSTEMI versus STEMI has been increasing globally, the prognostic impact of NSTEMI presentation may be even more important in the current era. Indeed, it is conceivable that the present study population represents a contemporary PCI population, with the use of intravascular ultrasound, drug-eluting stents, and MCS in 98.0%, 87.2%, and 56.6%, respectively, which is in line with current clinical practice patterns in Japan [32,33,34,35,36,37,38,39].

### 4.2. Ejection Fraction in Cardiogenic Shock

In contrast to types of MI, LVEF is often incorporated as a predictive factor in previous risk scoring systems [12]. A recent review paper introduced previously proposed risk scoring systems in a setting of acute MI and/or CS, including the IABP-SHOCK II score, CardShock score, APACHE score, simplified acute physiology score, TIMI risk score, PAMI score, Zwolle score, early risk stratification, SAVE score, ENCOURAGE mortality risk score, CS4P score, and others [12]. The CardShock score has seven items (age > 75 years, obnubilation, previous MI or coronary artery bypass grafting, ACS as an etiology of CS, low LVEF, lactic acid level, and renal function) to predict in-hospital mortality in patients with CS, in which LVEF < 40% is listed as a predictor [27]. Another scoring system reported by Garcia-Alvarez et al. includes only four items, one of which is LVEF < 25% [40]. In the present study, LVEF < 33% on admission was identified to be the best predictor. Taken together, although the threshold of LVEF is yet to be determined, it is conceivable that lower LVEF on admission leads to higher mortality in patients with acute MI with CS. On the other hand, when a patient with acute MI has preserved LVEF (≥ 33%), in-hospital mortality would be relatively low even when complicated by CS. Patients with LVEF < 33% in this study had exceedingly high in-hospital mortality with NSTEMI presentation (i.e., 66.7%). Although whether immediate invasive treatment including primary PCI and MCS improves clinical outcomes in NSTEMI patients with low LVEF is largely unknown, aggressive therapeutic strategies may be considered, as were performed in the present cohort.

### 4.3. Study Limitations

There are some limitations in the present study. This was a retrospective study with a modest sample size, and thus, further large-scale studies are warranted to externally confirm our results. Interventional procedures and medical treatment were left to treating physicians in a setting of real-world practice. Although several risk predicting models include lactic acid as a predicting factor [12], the data were not available in the present study. It is reported that shorter symptom-onset-to-balloon time is associated with better clinical outcomes in patients with STEMI [41], but the data are also lacking because of the retrospective nature of this study. Furthermore, older age and female gender have been identified as factors associated with poor prognosis in patients with acute MI complicated by CS in previous reports [12]; both were included in the multivariable model in this study but were not identified as significant factors. It is well known that women with acute MI were unlikely to undergo coronary revascularization as compared with men [42], while all patients received primary PCI in the present study population, which may explain the neutral effect of gender in this study. In addition, given that older patients are likely to present with NSTEMI rather than STEMI, the prognostic impact of NSTEMI presentation in this study may in part be driven by the higher age in this subset, although NSTEMI presentation was adjusted by age in the present study.

## 5. Conclusions

Even in a contemporary cohort of patients with acute MI undergoing PCI, in-hospital mortality remained high when complicated by CS. NSTEMI and lower LVEF were identified as factors associated with in-hospital death in this catastrophic condition. The identification of even higher-risk subsets and targeted therapeutic strategies may be needed to improve survival of patients with acute MI and CS.

## Figures and Tables

**Table 1:** Baseline characteristics.

Variable	All (*n* = 196)	Survivors (*n* = 119)	Non-Survivors (*n* = 77)	*p* Value
Age (years)	67.2 ± 11.5	66.8 ± 11.6	67.8 ± 11.3	0.58
Men	153 (78.1%)	89 (74.8%)	64 (83.1%)	0.16
Body mass index (kg/m^2^)	24.1 ± 3.6	24.2 ± 3.5	23.9 ± 4.0	0.59
Hypertension	125 (64.1%)	83 (69.8%)	42 (55.3%)	0.04
Diabetes	87 (44.4%)	53 (44.5%)	34 (44.2%)	0.96
Dyslipidemia	81 (41.5%)	59 (49.6%)	22 (29.0%)	0.004
Current smoker	67 (34.5%)	47 (39.8%)	20 (26.3%)	0.05
Prior MI	20 (10.3%)	13 (10.9%)	7 (9.2%)	0.70
Prior PCI	17 (8.7%)	9 (7.6%)	8 (10.5%)	0.48
Prior CABG	2 (1.0%)	1 (0.8%)	1 (1.3%)	0.75
Prior heart failure	8 (4.1%)	2 (1.7%)	6 (8.0%)	0.03
Hemodialysis	8 (4.1%)	3 (2.5%)	5 (6.6%)	0.17
Creatinine (mg/dL)	1.47 ± 1.3	1.27 ± 0.9	1.78 ± 1.6	0.006
Hemoglobin (g/dL)	13.3 ± 2.4	13.6 ± 2.1	12.9 ± 2.7	0.03
Clinical presentation				0.02
STEMI	147 (75.0%)	96 (80.7%)	51 (66.2%)	
NSTEMI	49 (25.0%)	23 (19.3%)	26 (33.8%)	
Cardiac arrest	117 (59.7%)	69 (58.0%)	48 (62.3%)	0.54

CABG, coronary artery bypass grafting; MI, myocardial infarction; NSTEMI, non-ST-segment elevation myocardial infarction; PCI, percutaneous coronary intervention; STEMI, ST-segment elevation myocardial infarction.

**Table 2:** Procedural characteristics.

Variable	All (*n* = 196)	Survivors (*n* = 119)	Non-Survivors (*n* = 77)	*p* Value
Culprit vessel				<0.001
RCA	56 (28.6%)	46 (38.7%)	10 (13.0%)	
LMT/LAD	110 (56.1%)	57 (47.9%)	53 (68.8%)	
LCX	18 (9.2%)	11 (9.2%)	7 (9.1%)	
Undetermined	12 (6.1%)	5 (4.2%)	7 (9.1%)	
Three vessel disease	59 (30.1%)	35 (29.4%)	24 (31.2%)	0.79
Intravascular ultrasound	191 (98.0%)	115 (97.5%)	76 (98.7%)	0.59
Drug-eluting stents	171 (87.2%)	103 (86.6%)	68 (88.3%)	0.76
Mechanical circulatory support	111 (56.6%)	52 (43.7%)	59 (76.6%)	<0.001
IABP	90 (45.9%)	45 (37.8%)	45 (58.4%)	0.005
ECMO	60 (30.6%)	21 (17.7%)	39 (50.7%)	<0.001
Intravascular microaxial LVAD	3 (1.5%)	3 (2.5%)	0 (0%)	0.08
Intubation	156 (79.6%)	84 (70.6%)	72 (93.5%)	<0.001
Final TIMI flow grade				0.04
0	1 (0.5%)	0 (0%)	1 (1.3%)	
1	2 (1.0%)	1 (0.8%)	1 (1.3%)	
2	52 (26.5%)	24 (20.2%)	28 (36.4%)	
3	141 (71.9%)	94 (79.0%)	47 (61.0%)	

Intravascular microaxial LVAD represents Impella (Abiomed, Danvers, USA). ECMO, extracorporeal membrane oxygenation; IABP, intra-aortic balloon pump; LAD, left anterior descending artery; LCX, left circumflex; LMT, left main trunk; LVAD, left ventricular assist device; RCA, right coronary artery; TIMI, Thrombolysis in Myocardial Infarction.

**Table 3:** Factors associated with in-hospital death.

Variable	Univariable			Multivariable	
	OR (95% CI)	*p* Value		OR (95% CI)	*p* Value
Age (years)	1.01 (0.98–1.03)	0.57		1.02 (0.98–1.07)	0.28
Men	1.66 (0.80–3.43)	0.16		2.23 (0.74–6.72)	0.15
Body mass index (kg/m^2^)	0.98 (0.90–1.06)	0.59		1.03 (0.91–1.17)	0.65
Hypertension	0.54 (0.29–0.97)	0.04		0.75 (0.29–1.97)	0.56
Diabetes	0.98 (0.55–1.75)	0.96			
Dyslipidemia	0.41 (0.22–0.76)	0.004		0.61 (0.25–1.48)	0.28
Current smoker	0.54 (0.29–1.01)	0.05		0.42 (0.15–1.13)	0.08
Prior MI	0.83 (0.31–2.18)	0.69			
Prior heart failure	5.09 (0.999–25.90)	0.03		1.50 (0.19–11.80)	0.70
LVEF (%)	0.92 (0.89–0.94)	<0.001		0.92 (0.89–0.96)	<0.001
Hemodialysis	2.72 (0.63–11.74)	0.17			
Creatinine	1.43 (1.07–1.88)	0.005		1.12 (0.79–1.59)	0.50
Hemoglobin	0.87 (0.77–0.99)	0.03		0.89 (0.74–1.09)	0.26
NSTEMI presentation	2.13 (1.1.0–4.10)	0.02		2.71 (1.01–7.33)	0.048
Cardiac arrest	1.20 (0.67–2.16)	0.54			
Culprit in the LMT/LAD	2.40 (1.32–4.38)	0.004		2.01 (0.83–4.87)	0.12
Three vessel disease	1.09 (0.58–2.03)	0.79			
Final TIMI flow grade 0–2	2.40 (1.27–4.53)	0.007		2.33 (0.88–6.13)	0.09

CI, confidence interval; LAD, left anterior descending artery; LMT, left main trunk; LVEF, left ventricular ejection fraction; MI, myocardial infarction; NSTEMI, non-ST-segment elevation myocardial infarction; OR, odds ratio; TIMI, Thrombolysis in Myocardial Infarction.

## Data Availability

Not applicable.

## Supplementary Materials

The following supporting information can be downloaded at: https://www.mdpi.com/article/10.3390/life12101672/s1, Table S1: Baseline characteristics of the patients with and without CS.
