# Supplementary material for: Prognostic Factors of In-Hospital Mortality in Patients with Acute Myocardial Infarction Complicated by Cardiogenic Shock
Source: Life (Basel). 2022 Oct 21;12(10):1672. doi: 10.3390/life12101672 (PMC9604739; doi:10.3390/life12101672)
Supplement: Supplementary file 1 [file life-12-01672-s001.zip › Table S1.pdf]

Table S1. Baseline characteristics of the patients with and without CS

Variable	All (n=1102)	CS (n=196)	Non-CS (n=906)	p value
Age (years)	67.3±12.1	67.2±11.5	67.4±12.3	0.86
Men	846 (76.8%)	153 (78.1%)	693 (76.5%)	0.64
Body mass index (kg/m^2)	24.2±3.9	23.8±4.4	24.3±3.7	0.13
Hypertension	745 (67.7%)	125 (64.1%)	620 (68.4%)	0.24
Diabetes	411 (37.3%)	87 (44.4%)	324 (35.8%)	0.02
Dyslipidemia	675 (61.3%)	81 (41.5%)	594 (65.6%)	<0.001
Current smoker	367 (33.4%)	67 (34.5%)	300 (33.1%)	0.70
Prior MI	73 (6.6%)	20 (10.3%)	53 (5.9%)	0.03
Prior PCI	107 (9.7%)	17 (8.7%)	90 (9.9%)	0.60
Prior CABG	19 (1.7%)	2 (1.0%)	17 (1.9%)	0.38
Prior heart failure	29 (2.6%)	8 (4.1%)	21 (2.3%)	0.18
Hemodialysis	39 (3.6%)	8 (4.1%)	31 (3.4%)	0.65
Creatinine (mg/dl)	1.21±1.44	1.47±1.26	1.16±1.47	0.006
Hemoglobin (g/dl)	13.7±2.2	13.3±2.4	13.8±2.2	0.01
Clinical presentation				0.01
STEMI	744 (67.5%)	147 (75.0%)	597 (65.9%)	
NSTEMI	358 (32.5%)	49 (25.0%)	309 (34.1%)	
Cardiac arrest	135 (12.3%)	117 (59.7%)	18 (2.0%)	<0.001
Culprit vessel				0.003
RCA	341 (30.9%)	56 (28.6%)	285 (31.5%)	
LMT/LAD	557 (50.5%)	110 (56.1%)	447 (49.3%)	
LCX	169 (15.3%)	18 (9.2%)	151 (16.7%)	
Undetermined	35 (3.2%)	12 (6.1%)	23 (2.5%)	
Three vessel disease	239 (21.7%)	59 (30.1%)	180 (19.9%)	0.002
Intravascular ultrasound	1065 (96.7%)	191 (98.0%)	874 (96.5%)	0.30
Drug-eluting stents	1001 (90.8%)	171 (87.2%)	830 (91.5%)	0.004
Mechanical circulatory support	141 (12.8%)	111 (56.7%)	30 (3.3%)	<0.001
IABP	119 (10.8%)	90 (45.9%)	29 (3.2%)	<0.001

ECMO	63 (5.7%)	60 (30.6%)	3 (0.3%)	<0.001
Intravascular microaxial LVAD	3 (0.3%)	3 (1.5%)	0 (0%)	0.001
Intubation	185 (16.8%)	156 (79.6%)	29 (3.2%)	<0.001
Final TIMI flow grade				<0.001
0	9 (0.8%)	1 (0.5%)	8 (0.9%)	
1	10 (0.9%)	2 (1.0%)	8 (0.9%)	
2	141 (12.8%)	52 (26.5%)	89 (9.8%)	
3	942 (85.5%)	141 (71.9%)	801 (88.4%)	

Cardiac arrest is before or on admission. CABG, coronary artery bypass grafting; ECMO, extracorporeal membrane oxygenation; IABP, intra-aortic balloon pump; LAD, left anterior descending artery; LCX, left circumflex; LMT, left main trunk; LVAD, left ventricular assist device; MI, myocardial infarction; NSTEMI, non-ST-segment elevation myocardial infarction; PCI, percutaneous coronary intervention; RCA, right coronary artery; STEMI, ST-segment elevation myocardial infarction; TIMI, Thrombolysis In Myocardial Infarction.
